# Recent Advances in Targeting CD8 T-Cell Immunity for More Effective Cancer Immunotherapy

**DOI:** 10.3389/fimmu.2018.00014

**Published:** 2018-01-22

**Authors:** Aurélie Durgeau, Yasemin Virk, Stéphanie Corgnac, Fathia Mami-Chouaib

**Affiliations:** ^1^INSERM UMR 1186, Integrative Tumor Immunology and Genetic Oncology, Gustave Roussy, EPHE, Fac. de Médecine – Univ. Paris-Sud, Université Paris-Saclay, Villejuif, France; ^2^ElyssaMed, Paris Biotech Santé, Paris, France

**Keywords:** immunotherapy of cancer, cytotoxic T lymphocytes, tumor antigens, neoantigens, T-cell receptor repertoire

## Abstract

Recent advances in cancer treatment have emerged from new immunotherapies targeting T-cell inhibitory receptors, including cytotoxic T-lymphocyte associated antigen (CTLA)-4 and programmed cell death (PD)-1. In this context, anti-CTLA-4 and anti-PD-1 monoclonal antibodies have demonstrated survival benefits in numerous cancers, including melanoma and non-small-cell lung carcinoma. PD-1-expressing CD8^+^ T lymphocytes appear to play a major role in the response to these immune checkpoint inhibitors (ICI). Cytotoxic T lymphocytes (CTL) eliminate malignant cells through recognition by the T-cell receptor (TCR) of specific antigenic peptides presented on the surface of cancer cells by major histocompatibility complex class I/beta-2-microglobulin complexes, and through killing of target cells, mainly by releasing the content of secretory lysosomes containing perforin and granzyme B. T-cell adhesion molecules and, in particular, lymphocyte-function-associated antigen-1 and CD103 integrins, and their cognate ligands, respectively, intercellular adhesion molecule 1 and E-cadherin, on target cells, are involved in strengthening the interaction between CTL and tumor cells. Tumor-specific CTL have been isolated from tumor-infiltrating lymphocytes and peripheral blood lymphocytes (PBL) of patients with varied cancers. TCRβ-chain gene usage indicated that CTL identified *in vitro* selectively expanded *in vivo* at the tumor site compared to autologous PBL. Moreover, functional studies indicated that these CTL mediate human leukocyte antigen class I-restricted cytotoxic activity toward autologous tumor cells. Several of them recognize truly tumor-specific antigens encoded by mutated genes, also known as neoantigens, which likely play a key role in antitumor CD8 T-cell immunity. Accordingly, it has been shown that the presence of T lymphocytes directed toward tumor neoantigens is associated with patient response to immunotherapies, including ICI, adoptive cell transfer, and dendritic cell-based vaccines. These tumor-specific mutation-derived antigens open up new perspectives for development of effective second-generation therapeutic cancer vaccines.

## Introduction

CD8^+^ T lymphocytes play a central role in immunity to cancer through their capacity to kill malignant cells upon recognition by T-cell receptor (TCR) of specific antigenic peptides presented on the surface of target cells by human leukocyte antigen class I (HLA-I)/beta-2-microglobulin (β2m) complexes. TCR and associated signaling molecules thus become clustered at the center of the T cell/tumor cell contact area, resulting in formation of a so-called immune synapse (IS) (1) and initiation of a transduction cascade, leading to execution of cytotoxic T lymphocyte (CTL) effector functions. Major CTL activities are mediated either directly, through synaptic exocytosis of cytotoxic granules containing perforin and granzymes into the target, resulting in cancer cell destruction, or indirectly, through secretion of cytokines, including interferon (IFN)γ and tumor necrosis factor (TNF). Adhesion/costimulatory molecules, mainly lymphocyte-function-associated antigen-1 (LFA-1, CD11a/CD18 or α_L_/β_2_) and CD103 (α_E_/β_7_) integrins, on CTL play a critical role in TCR-mediated killing by interacting with their cognate ligands, intercellular adhesion molecule 1 (or CD54) and E-cadherin, respectively, and directing exocytosis of lytic granules to the cancer cell surface at the IS (2, 3). NKG2D, a c-type lectin molecule expressed on activated lymphocytes (4, 5), also plays an important role in the induction of T-cell-mediated cytoxicity and in CTL-dependent rejection of cancer (6, 7). NKG2D ligands include major histocompatibility complex class I-related chain (MIC)A and MICB (8), and UL16-binding proteins 1, 2, and 3 (9). These ligands are upregulated upon cell stress, such as tumor transformation, and are expressed by most of the cancer cells (10) in particular those of epithelial origin (11).

Activation of naive CD8 T cells by antigen-presenting cells (APC) involves binding of TCR, that is associated with the CD3 complex, to specific peptide-major histocompatibility complex class I (pMHC-I) complexes and the interaction of the costimulatory molecules CD28 and CD2 with their respective ligands CD80/CD86 and LFA-3 (12). Costimulatory receptors such as TNF receptor family member 4 (TNFRSF4 best known as OX40 or CD134) and member 9 (TNFRSF9 best known as 4-1BB or CD137) also play an important role in T-cell priming and antitumor immune responses (13–17).

## Antitumor T-Cell Responses

Evidence for antitumor CD8^+^ T-cell immunity was provided by isolation of tumor-specific CTL from peripheral blood or tumor tissue of patients with diverse cancers, such as melanoma and lung carcinoma (18–22). The existence of a tumor-specific CTL response was further strengthened by identification of tumor-associated antigens (TAA) and detection of TAA-specific CD8^+^ T cells in spontaneously regressing tumors (18). Moreover, a correlation between tumor progression control and the infiltration rate of CD8^+^ T lymphocytes in the tumor was established (23). Efficacy of the antitumor immune response is negatively influenced by a hostile tumor microenvironment. Establishment of an immunosuppressive state within the tumor is mediated by diverse immunosuppressive factors released by cancer cells themselves, such as vascular endothelial growth factor, transforming growth factor-β (TGF-β) and indoleamine 2,3-dioxygenase, and/or by recruiting regulatory immune cells with immunosuppressive functions, such as regulatory T (Treg) cells and myeloid-derived suppressor cells (MDSC) (24). Indeed, a role for Treg cells in modulating tumor-specific effector T lymphocytes by producing immunosuppressive cytokines, such as IL-10 and TGF-β, consuming IL-2 or expressing the inhibitory molecule cytotoxic T-lymphocyte associated antigen (CTLA)-4, has been reported (25, 26). MDSC are a heterogeneous group of myeloid progenitor cells and immature myeloid cells, including immature macrophages, granulocytes, and dendritic cells (DC), that impair T-lymphocyte functions by upregulating the expression of immune suppressive factors, such as arginase and inducible nitric oxide synthase, increasing the production of nitric oxide (NO) and reactive oxygen species, and inducing Treg cells (27). Moreover, it has been shown that predominant secretion of TNF by CD4^+^ T cells in MHC class II-expressing melanoma promotes a local immunosuppressive environment, impairing effector CD8^+^ T-cell functions (28).

While it is generally admitted that CD8^+^ T cells are directly involved in antitumor cytotoxic responses, the role of CD4^+^ T cells is more controversial. Involvement of CD4^+^ T cells in regulating antitumor immunity was associated with their help in priming of CD8^+^ T cells, through activation of APC and an increase in antigen presentation by major histocompatibility complex class I (MHC-I) molecules *via* secretion of cytokines such as IFNγ (29, 30). More recently, it has been shown that CD4^+^ T-cell help optimized CTL in expression of cytotoxic effector molecules, downregulation of inhibitory receptors, and increased migration capacities (31). A role for the CD4^+^ T-cell subset in optimizing the antitumor immune response was supported by *in vivo* studies demonstrating that depletion of CD4^+^ T lymphocytes promotes tumor progression, whereas their adoptive transfer was correlated with improved tumor regression (32). Moreover, it has been reported that CD4^+^ T cells recognize most tumor-specific immunogenic mutanomes, and that vaccination with such CD4^+^ immunogenic mutations confers antitumor activity and broadens CTL responses in mice (33). Frequent recognition of neoantigens by CD4^+^ T cells was also observed in human melanoma (34). Notably, CD4^+^ CTL able to kill specific tumor cells have been described in several cancer types, including non-small-cell lung carcinoma (NSCLC), cutaneous T-cell lymphoma, and melanoma (35–39); for review, see Ref. (32). Elsewhere, TAA-specific CD4^+^ T-cell clones were shown to mediate HLA-II-restricted cytotoxic activity, making them attractive effectors in cancer immunotherapy (39, 40). While CD4^+^ CTL are able to lyse target cells *via* the granule exocytosis pathway (35, 36, 41, 42), they mainly use FasL- and APO2L/TRAIL-mediated pathways to kill their target cells (35, 43).

## Tumor Antigens Recognized by T Cells

Our fundamental knowledge of the tumor-specific T-cell response came with the discovery of tumor antigens that differentiated malignant cells from their non-transformed counterparts and provided important input in the field of tumor immunology and cancer immunotherapy. The first human tumor antigen recognized by CTL was identified in melanoma and was designated melanoma-associated antigen (MAGE)-1 (44). Subsequently, several other antigens of the MAGE family were characterized, most of which were identified through generation of tumor cell lines and isolation of reactive autologous CTL clones. Based on their expression profile, tumor antigens were initially classified into two categories: TAA and tumor-specific antigens (TSA). TAA are relatively restricted to tumor cells, and, to a limited degree, to normal tissues, whereas TSA are expressed only in tumor cells, arising from mutations that result in novel abnormal protein production.

At present, numerous TAA have been identified in a large variety of human cancer types. They are heterogeneous in nature and were classified into at least four groups according to their expression repertoire and the source of the antigen: antigens encoded by cancer-germline genes, differentiation antigens, overexpressed antigens, and viral antigens (Table 1). Antigens encoded by cancer-germline genes are expressed in tumor cells and in cells from adult reproductive tissues, including placenta and testicular cells, and are thus designated cancer testis antigens. Differentiation antigens are expressed only in tumor cells and in the normal tissue of origin, while overexpressed antigens are derived from proteins that are overexpressed in tumors, but are expressed at much lower levels in normal tissues. Viral antigens derive from viral infection and are associated with several human cancers, including cervical carcinoma, hepatocarcinoma, nasopharyngeal carcinoma, and adult T-cell leukemia (45, 46).

**Table 1:** Classification of tumor-associated antigens.

Type of antigens	Antigen characteristics	Example of human tumor antigens
Cancer-germline	Expressed only by tumor cells and adult reproductive tissues	MAGE, BAGE, GAGE, NY-ESO-1
Differentiation	Expressed by tumors and a limited range of normal tissues	Tyrosinase, Melan-A, gp100, CEA, MART-1
Overexpressed	Expressed by both normal and tumor cells, but much highly expressed in tumor cells	HER2, WT1, MUC1, ppCT
Viral	Expressed only by tumor cells as a result of viral infection	HPV, HBV, EBV, HTLV

The first mutant TSA, also termed neoantigens, were identified by the genetic method (46) *via* isolation of reactive CD8^+^ and CD4^+^ T-cell clones (Table 2). Recent accessibility to next-generation sequencing (NGS) technology and improvement in *in silico* epitope prediction have contributed to identification of patient-specific tumor antigens generated by somatic mutations in individual tumors (Table 3). Notably, most mutations identified in tumor-expressed genes do not generate neoantigens recognized by cognate T lymphocytes. Moreover, a large fraction of these mutations are not shared between patients and may thus be considered patient specific (47). These neoantigens have opened up new perspectives in cancer immunotherapy. They were shown to be involved in the success of immune checkpoint inhibitor (ICI) (48–50), adoptive cell transfer (ACT) immunotherapy (51, 52), and even virally induced epithelial cancer (53) and DC-based immunotherapy (54, 55); thus, they might be of use as predictive biomarkers of the response to immunotherapy.

**Table 2:** Mutant tumor antigens recognized by CD8 or CD4 T cells.

Gene/protein	Tumor type	Human leukocyte antigen (HLA)	Peptide	Position	Reference
		
		Class I	CD8 T-cell epitope		
LPGAT1	Bladder tumor	B44	AEPINIQTW	262–270	(56)
CASP-8	Head and neck SCC	B35	FPSDSWCYF	476–484	(57)
Beta-catenin	Melanoma	A24	SYLDSGIHF	29–37	(58)
CDK4	Melanoma	A2	ACDPHSGHFV	23–32	(59)

CDKN2A	Melanoma	A11	AVCPWTWLRG	125–133 (p14ARF-ORF3)	(60)
HLA-A11d	Melanoma					

CLPP	Melanoma	A2	ILDKVLVHL	240–248	(61)

GPNMB	Melanoma	A3	TLDWLLQTPK	179–188	(62)
RBAF600	Melanoma	B7	RPHVPESAF	329–337	
SIRT2	Melanoma	A3	KIFSEVTLK	192–200	
SNRPD1	Melanoma	B38	SHETVIIEL	11–19	
SNRP116	Melanoma	A3	KILDAVVAQK	668–677	

MART2	Melanoma	A1	FLEGNEVGKTY	446–455	(63)

MUM-1f	Melanoma	B44	EEKLIVVLF	30–38	(64)

MUM-2	Melanoma	B44	SELFRSGLDSY	123–133	(65)
		Cw6	FRSGLDSYV	126–134	

MUM-3	Melanoma	A68	EAFIQPITR	322–330	(66)
Myosin class I	Melanoma	A3	KINKNPKYK	911–919	(67)
N-ras	Melanoma	A1	ILDTAGREEY	55–64	(68)
OS-9	Melanoma	B44	KELEGILLL	438–446	(69)
Elongation factor 2	Lung SCC	A68	ETVSEQSNV	581–589	(70)
NFYC	Lung SCC	B52	QQITKTEV	275–282	(71)
Alpha-actinin-4	NSCLC	A2	FIASNGVKLV	118–127	(72)
Malic enzyme	NSCLC	A2	FLDEFMEGV	224–232	(20)
HLA-A2	RCC				(73)
Hsp70-2	RCC	A2	SLFEGIDIYT	286–295	(74)

		**Class II**	**CD4 T-cell epitope**		

COA-1	CRC	DR4	TLYQDDTLTLQAAGE	447–46	(75)
		DR13			

ARTC1	Melanoma	DR1	YSVYFNLPADTIYTNH		(76)
CDC27	Melanoma	DR4	FSWAMDLDPKGAE	760–771	(77)
FN1	Melanoma	DR2	MIFEKHGFRRTTPP	2050–2063	(78)

LDLR-FUT fusion protein	Melanoma	DR1	WRRAPAPGA	315–323	(79)
			PVTWRRAPA	312–320	

neo-PAP	Melanoma	DR7	RVIKNSIRLTLE	724–734	(80)
PTPRK	Melanoma	DR10	PYYFAAELPPRNLPEP	667–682	(81)
Triosephosphate isomerase	Melanoma	DR1	GELIGILNAAKVPAD	23–37	(82)

*SCC, squamous cell carcinoma; RCC, renal cell carcinoma; CRC, colorectal carcinoma; NSCLC, non-small-cell lung carcinoma*.

*From: https://www.cancerresearch.org/scientists/events-and-resources/peptide-database (slightly modified)*.

**Table 3:** Validated mutant antigens identified by WES and recognized by CD8 T cells.

Gene/protein	Tumor	Human leukocyte antigen	Peptide	Position	Reference
SETDB1	Cervival cancer	B40	VESEDIAEL	17–25	(53)
METTL17	Cervival cancer	A32	RTKVVQTLW	277–285	
ALDH1A1	Cervival cancer	B35	IPIDGIFFT	66–74	

CDKN2A	Melanoma	A2	KMIGNHLWV	153–161	(55)
TKT	Melanoma	A2	AMFWSVPTV	435–443	
TMEM48	Melanoma	A2	CLNEYHLFL	161–169	
AKAP13	Melanoma	A2	KLMNIQQKL	278–286	
OR8B3	Melanoma	A2	QLSCISTYV	186–194	
SEC24A	Melanoma	A2	FLYNLLTRV	465–473	
EXOC8	Melanoma	A2	IILVAVPHV	649–658	
MRPS5	Melanoma	A2	HLYASLSRA	58–66	
PABPC1	Melanoma	A2	MLGEQLFPL	516–524	

KIF2C	Melanoma	A2	RLFPGLTIKI	10–19	(52)
POLA2	Melanoma	Cw7	TRSSGSHFVF	413–422	

CCT6A	Melanoma	B27	LRTKVYAEL	156–164	(54)
TRRAP	Melanoma	A2	LLYQELLPL	774–782	
DNMT1	Melanoma	A24	IYKAPCENW	835–843	
PABPC3	Melanoma	A24	YYPPSQIAQL	416–425	
MAGE-A10	Melanoma	A24	LYNGMEHLI	255–263	
FMN2	Melanoma	A3	HSVSSAFKK	843–851	
WASL	Melanoma	B7	YPPPPPALL	343–351	

MAGEA6	Melanoma	A1	KVDPIGHVY	168–176	(83)
		B15	LMKVDPIGHVY	166–176	
		Cw5	KVDPIGHVYF	168–177	

PDS5A	Melanoma	Cw3	FVVPYMIYLL	1000–1009	

MED13	Melanoma	A1	VSVQIISCQY	1685–1694	
		A30	VQIISCQY	1687–1694	
		B15			

FLNA	Melanoma	B7	CVRVSGQGL	2049–2057	
KIB1B	Melanoma	B7	APARLERRHSA	1009–1018	

KFI1BP	Melanoma	A24	AYHSIEWAI	243–251
		B38	YHSIEWAI	244–251
		Cw12	NAYHSIEWAI	242–251

NARFL	Melanoma	A3	KSQREFVRR	62–70	(84)
PPFIA4	Melanoma	B39	MRMNQGVCC	706–714	
CDC37L1	Melanoma	A2	FLSDHLYLV	181–189	
MLL3	Melanoma	B7	KPSDTPRPVM	1026–1035	
FLNA	Melanoma	A2	HIAKSLFEV	364–372	
		B44	AGQHIAKSLF	361–370	
DOPEY2	Melanoma	B7	KPFCVLISL	362–370	
TTBK2	Melanoma	B7	RPHHDQRSL	1174–1182	
KIF26B	Melanoma	A11	SSYTGFANK	254–263	
SPOP	Melanoma	A2	FLLDEAIGL	141–149	
CDK4	Melanoma	A2	ALDPHSGHFV	23–32	
RETSAT	Melanoma	A68	HSCVMASLR	545–553	
		B37	HDLGRLHSC	539–547	
CLINT1	Melanoma	B57	VSKILPSTW	469–477	
COX7A2	Melanoma	A11	GVADVLLYR	80–88	

FAM3C	Melanoma	B44	TESPFEQHI	192–200	(48)
CSMD1	Melanoma		GLEREGFTF		

PPP1R3B	Melanoma	A1	YTDFHCQYV	172–180	(85)
CDK12	Melanoma	A11	CILGKLFTK	924–932	
CSNK1A1	Melanoma	A2	GLFGDIYLA	26–34	
GAS7	Melanoma	A2	SLADEAEVYL	141–150	
MATN	Melanoma	A11	KTLTSVFQK	226–234	
HAUS3	Melanoma	A2	ILNAMIAKIJ	154–162	

MTFR2	Non-small-cell lung carcinoma (NSCLC)		FAFQEYDSF	321–326	(50)
CHTF18	NSCLC		LLDIVAPK	765–772	
MYADM	NSCLC		SPMIVGSPW	22–30	

HERC1	NSCLC	A11	ASNASSAAK	3274–3282	(49)
HSDL1	Ovarian cancer	Cw14	CYMEAVAL	20–27	(86)

## Processing of CD8 T-Cell Epitopes

Most antigenic peptides recognized by CD8^+^ T cells originate from degradation of intracellular proteins by proteasomes and translocation to the lumen of the endoplasmic reticulum (ER) by the transporter associated with antigen processing (TAP)1/TAP2 heterodimeric complex. Once in the ER, peptides larger than 11 residues are further cleaved by ER amino-peptidase (ERAP)1 and ERAP2 before being loaded onto MHC-I molecules and presented on the surface of target cells for CD8 T-cell recognition [for review, see Ref. (87, 88)].

Defects in the antigen-processing machinery and, in particular, in TAP subunits, have been described as a major mechanism used by several tumors to escape from CD8 T-cell immunity (89). In this context, alternative peptide degradation pathways permitting CD8 T cells to overcome this tumor evasion mechanism have been identified. Indeed, proteasome/TAP-independent CTL epitopes, generated either by the cytosolic metallopeptidase insulin-degrading enzyme or cytosolic endopeptidases nardilysin and thimet oligopeptidase, have been described (90, 91). Moreover, TAP-independent processing of antigenic peptides can be achieved by the so-called secretory pathway in which the proteolytic enzyme furine releases C-terminal peptides (92). Interestingly, peptide epitopes that emerge at the surface of cancer cells with impaired TAP function derived from self-antigens and act as immunogenic neoantigens, as they are not presented by normal cells (93). Our group identified a signal peptide-derived CD8 T-cell epitope processed independently of proteasomes/TAP, by a novel pathway involving signal peptidase and the signal peptide peptidase (94, 95). These signal sequence-derived peptides represent attractive T-cell targets that permit CTL to destroy TAP-impaired tumors and therefore correspond to promising candidates for cancer immunotherapy.

## The TCR Repertoire and Antitumor T-Cell Immunity

The TCR–CD3 complex, expressed on the T-cell surface, allows recognition of antigenic peptides bound to MHC molecules on target cells and APC, and transduction of the signal into the cytosol to initiate signaling events leading to T-cell activation (96). The TCRα- and β-chains are products of V(D)J recombination, a somatic rearrangement of the germline TCR loci occurring in T cells (97). This process leads to generation of a diverse TCR repertoire [>10^15^ distinct αβ-receptors or clonotypes (98)] that enables T-cell recognition of numerous foreign or mutant antigens. The TCRα- and β-chains possess three hypervariable regions, referred to as complementarity-determining regions (CDR) 1, 2, and 3. CDR3 is highly polymorphic and is directly responsible for recognition of antigenic peptides. Immunoscope/spectratype technology was first used to probe the T-cell repertoire by analyzing the diversity of TCRVβ (99, 100) and, more recently, TCRVα (101, 102) chains without isolating peptide-reactive T cells and cloning TCR genes. It is based on the use of V and J gene-segment-specific primers for reverse transcription-polymerase chain reaction amplification of CDR3 of a bulk T-cell population from diverse biological materials such as blood and tumor tissues (103). Analyzing CDR3 polymorphisms and sequence length diversity served to follow up T-cell clonality in tumor-infiltrating lymphocytes (TIL) to investigate T-cell functions and the pattern of TCR utilization. It highlighted restriction of the CDR3 length of TCRβ- and TCRα-chains in T cells infiltrating solid tumors and hematological malignancies, including melanoma, renal cell carcinoma (RCC), neuroblastoma, NSCLC, and Sezary syndrome (19, 101, 104–109). TCRβ-chain gene usage also showed that antigen-specific T-cell clones with high functional avidity/tumor reactivity expanded only at the tumor site, but not in peripheral blood (108). Identification of TAA has led to improvement in procedures for detecting and monitoring specific antitumor T-cell responses. In this regard, combining a quantitative immunoscope approach with MHC–peptide multimer-based T-cell sorting led to more sensitive *ex vivo* follow-up, by quantitation of human CD8^+^ T-cell responses and monitoring of T-cell subsets throughout immunotherapy clinical trials (110).

Tremendous progress in characterizing the size and dynamics of the T-cell repertoire has emerged from recent advances in DNA and RNA sequencing (RNAseq) technologies (111, 112). High-throughput TCR sequencing (TCR-seq) involves NGS for generating DNA sequences covering TCR CDR3 and permits quantification of T-cell diversity at very high resolution (113). Another method for profiling the TCR repertoire relies on a TCR-specific short read assembly strategy based on 5′ amplification of cDNA ends (RACE), so as to obtain TCRβ CDR3 transcript sequences and massively parallel Illumina sequencing of TCRβ CDR3 amplification products (114). This strategy avoids potential bias associated with the use of multiple primer sets required to amplify CDR3 regions from all *TCRBV* sequences and takes advantage of the conserved sequences of *TCRBC1* and *TCRBC2* genes (115, 116). High-throughput DNA-based strategy for identifying antigen-specific TCR sequences was also developed by the capture and sequencing of genomic DNA fragments encoding TCR genes (117). More recently, an optimized approach to characterizing tissue-resident T-cell (T_RM_) populations emerged from extraction of TCR CDR3 sequence information directly from RNAseq data sets of thousands of solid tumors and control tissues (118). This method circumvents the need for PCR amplification and provides TCR information in the context of global gene expression profiles.

Sequence-based immunoprofiling is a useful tool for monitoring the dynamics of the T-cell repertoire under physiological and pathological conditions, and in response to therapeutic interventions. In this respect, characterization of the TCR repertoire in TIL permits isolation of tumor-specific T-cell clones for use in cancer immunotherapy. TCR-seq can also be used to evaluate T-cell diversity and identify tumor-reactive T-cell clonotypes, along with potentially immunogenic neoantigen-reactive T cells (119). For instance, deep cDNA sequencing of TCR-α and β-chains enabled quantitative monitoring of the T-cell repertoire in lung cancer patients treated with cancer peptide vaccines (120). Another interesting parameter for follow-up by deep TCR-seq is the heterogeneity of T-cell density and clonality across tumor regions. Indeed, it has been shown that high intratumor heterogeneity of TCR is positively correlated with that of predicted neoantigens and has been associated with increased risk of disease progression (121). In contrast, maintenance of high-frequency TCR clonotypes alongside CTLA-4 blockade therapy was associated with improved overall survival in prostate cancer and melanoma (122). Moreover, high TCR clonality was associated with an increased response by melanoma patients to the programmed cell death (PD)-1 blockade, suggesting that TCR repertoire analysis could be used as a predictive marker in cancer immunotherapy (123). Indeed, elevated TCR clonality and significant T-cell clone expansion were observed in melanoma patients responding to anti-PD1 treatment (124). Overall, T-cell clonality and TCR repertoire diversity appear to be biomarkers of antitumor adaptive immunity and might also be predictive markers of responses to cancer immunotherapy.

## T-Cell-Based Cancer Immunotherapies

An understanding of regulation of the molecular interaction between T cells and tumor cells, together with refined T-cell engineering technologies and the discovery of TSA, gave rise to novel cancer immunotherapies with unprecedented clinical efficacy. These therapies are aimed at (re)activating and expanding tumor-specific CTL, with the goal of destroying primary cancer cells and metastases. The most effective current cancer immunotherapies include ICI, such as anti-PD-1 and anti-CTLA-4, ACT of *ex vivo*-expanded tumor-reactive T cells, either native (CTL clones or TIL) or engineered to express particular TCR or chimeric antigen receptors (CAR), and TSA-based cancer vaccines (peptide- or RNA-based) (84, 125–132). Moreover, increasing evidence of a link between CD8 and CD4 T-cell recognition of mutant neoepitopes and clinical responses to cancer immunotherapy strategies has been reported (34, 48–53, 55); for review, see Ref. (47).

### ACT Immunotherapy

The possibility of expanding subsets of mature T cells *in vitro* led to development of ACT immunotherapy. The aim is to transfer a T-cell population enriched in potentially highly tumor-reactive effector cells (130, 131, 133, 134). In this context, re-infusion of *ex vivo*-expanded TIL displaying increased specificity toward cancer cells was developed as a means of strengthening patient spontaneous T-cell responses and overcoming tolerance to the tumor. Steven Rosenberg’s team has been one of the pioneer*s* in the development of ACT, mainly using selected tumor-reactive T cells and TIL. Thus, clonal repopulation of T cells directed against overexpressed self-derived differentiation antigens, in combination with chemotherapy and high doses of IL-2, led to tumor regression in patients with metastatic melanoma (135, 136). Similarly, treatment of patients with uveal melanoma by adoptive transfer of autologous TIL, administered together with IL-2, resulted in objective tumor regression (137). Clinical responses were associated with the presence of tumor-resident CD8^+^ T lymphocytes that target tumor-specific mutant neoantigens and express the PD-1 checkpoint receptor (51, 52, 83, 138, 139). Moreover, neoantigen-reactive TCR have been identified from the most frequent clonotypes among TIL, opening up new avenues for developing a personalized TCR-gene therapy approach that targets individual sets of antigens presented by tumor cells without the need for determining their identity (140). Accordingly, neoantigen-reactive TCR have been identified, with the aim of treating patients with autologous T cells genetically modified to express such TCR (141). Nevertheless, analyses of neoantigen-specific T-cell responses in melanoma patients treated by ACT demonstrated that the T-cell-recognized neoantigens can be selectively lost over time emphasizing the importance of targeting broad TCR recognized neoantigens to avoid tumor resistance (142).

While ACT of tumor-specific T cells holds promise for melanoma treatment, significant challenges remain in clinical translation to other solid tumors. This can be explained by the observation that some tumors, referred to as “immune-desert tumors” or “cold tumors,” are rarely infiltrated by T cells, and TIL often display an exhausted state acquired in the tumor microenvironment. Indeed, TIL are characterized by high expression levels of one or several inhibitory receptors such as PD-1, CTLA-4, Tim-3, LAG-3, and TIGIT, and often display altered production of cytokines leading to weak antitumor reactivity (143, 144); for review, see Ref. (145). Moreover, the limited life span of TIL and difficulties linked to their production, including isolation from fresh patient tumor specimens and selection based on tumor-specificity, constrain their clinical routine use.

To overcome limitations of TIL-based ACT, and due to the availability of TAA-specific TCR or antibodies, genetically engineered T cells have been developed with either tumor-specific TCR or CAR (146–149). Therefore, desired specificity was achieved by genetically modifying T cells to express a TAA-specific TCR (150–153). Candidates are selected either from the native TCR repertoire or after mutagenesis of their antigen recognition domain, the CDR3 domain, to increase the affinity of T cells (154). Thus, T cells engineered to express TAA-specific TCR (recognizing Melan-A/MART1-, gp100-, NY-ESO-, or p53-derived peptides) resulted in objective regression of metastatic melanoma lesions in some patients (153, 155, 156). As an alternative, engineered T-cell strategy utilizes CAR comprising the antigen-binding domain of an antibody, fused with one or more immunostimulatory domains, to activate T cells once the recognition domain has bound to a target cell. Because such T cells are able to recognize tumor antigen-expressing cells in a MHC-independent manner, a single CAR can be used on all patients whose tumor expresses the target antigen (i.e., CD19, CD20). The therapeutic potential of CAR-expressing T cells, especially in patients with hematological malignancies such as B-cell lymphoma expressing CD19 or CD20, has been demonstrated in several clinical trials (157–163). This holds promise for further use in hematological tumors and for treatment of solid tumors unresponsive to other immunotherapies.

### Immune Checkpoint Blockade Immunotherapy

Targeting immune checkpoints with blocking monoclonal antibodies (mAb) such as anti-CTLA-4 and anti-PD-1 or anti-PD-L1 has provided clinical benefits for patients with advanced metastatic melanoma, NSCLC, RCC, and several other cancers (164, 165). While the CTLA-4 blockade reduces the activation threshold required for T-cell priming (166), the PD1/PD-L1 blockade in certain T-cell subpopulations (167) at least partly reverses immune alterations such as exhaustion (168). This allows synergy for combined treatments (169) and opens up new perspectives for combining these checkpoint blockers (i.e., anti-CTLA-4, -PD-1, or -PD-L1) with mAb toward additional inhibitory molecules, such as BTLA, TIM-3, or LAG-3. In this regard, synergistic antitumor effects were obtained in several preclinical models (170–172).

Accumulating evidence indicates that preexisting antitumor CD8^+^ T cells predict the efficacy of ICI therapy (124, 173). Moreover, effective CTLA-4 and PD-1 blockade immunotherapy appears to be associated with the presence of T cells directed toward mutant cancer neoepitopes (48–50), and with the likelihood of MHC presentation of these neoantigens and subsequent recognition by specific T cells (174). Mutant neoantigens are highly immunogenic; they are not expressed by normal tissues and thus bypass thymic tolerance (175). Unfortunately, clinical trials demonstrated that only a fraction of cancer patients respond to such immunotherapy. Resistance to anti-PD-1 of tumors with a high mutational load was associated with defects in pathways involved in IFNγ-receptor signaling and antigen presentation by MHC-I molecules, concomitant with a truncating mutation in the gene encoding β2m (176, 177). Moreover, patients identified as non-responders to anti-CTLA-4 mAb had tumors with genomic defects in IFN-γ pathway genes (178). These findings demonstrate the importance of the IFN-γ signaling pathway and CD8 T-cell recognition of mutant neoantigens in response to checkpoint blockade immunotherapy.

### Therapeutic Cancer Vaccines

The discovery of TAA has led to development of therapeutic cancer vaccines, based on either synthetic peptides, “naked” DNA, DC, or recombinant viruses, that attempt to strengthen the antitumor immune response, and particularly tumor antigen-specific CTL response (179, 180). Peptide vaccines have many advantages, including inexpensive, convenient acquisition of clinical-grade peptides, easy administration, higher specificity, and potency due to their higher compatibility with targeted proteins, the ability to penetrate the cell membrane and improved safety with few side effects (181, 182). Mechanisms underlying priming of anticancer immune responses by peptide-based vaccines, and hence their efficacy, is dependent, at least in part, on the size of the peptides. While short peptides (8–11 aa) bind directly to HLA-I molecules and mount MHC-I-restricted antigen-specific CD8^+^ T-cell immunity (183–185), long synthetic peptides (25–50 aa) must be taken up, processed, and presented by APC to elicit a T-cell response. Vaccination with long peptides usually results in broader immunity than with short peptides, along with induction of both CD8^+^ cytotoxic and CD4^+^ helper T cells when conjugated with efficient adjuvants (186, 187). Indeed, CD4^+^ T-cell help is required for generation of potent CTL and long-lived memory CD8^+^ T cells (186).

First-generation cancer vaccines based on non-mutant TAA, also termed shared antigens because they are expressed by many patients’ tumors, such as MART-1, gp100, tyrosinase, TRP-2, NY-ESO-1, MAGE-A3, and Her2/neu or telomerase proteins, were shown to be immunogenic and capable of inducing clinical responses in only a minority of patients with late-stage cancer (180, 188, 189). However, results showing that CD4^+^ T cells directed toward NY-ESO-1 cancer-germline TAA and lymphocytes genetically engineered with a NY-ESO-1-reactive TCR display antitumor activity (40, 190) support the notion that T-cell responses to a subset of non-mutant antigens contribute to the effects of current cancer immunotherapies. The limited success of these active immunotherapy approaches might be due to the inability of effector T cells to overcome tolerance to self-antigens, expression of T-cell inhibitory receptors such as CTLA-4 and PD-1, and suboptimal activation of tumor-specific T cells in an immunosuppressive tumor microenvironment (191).

**Figure 1:**
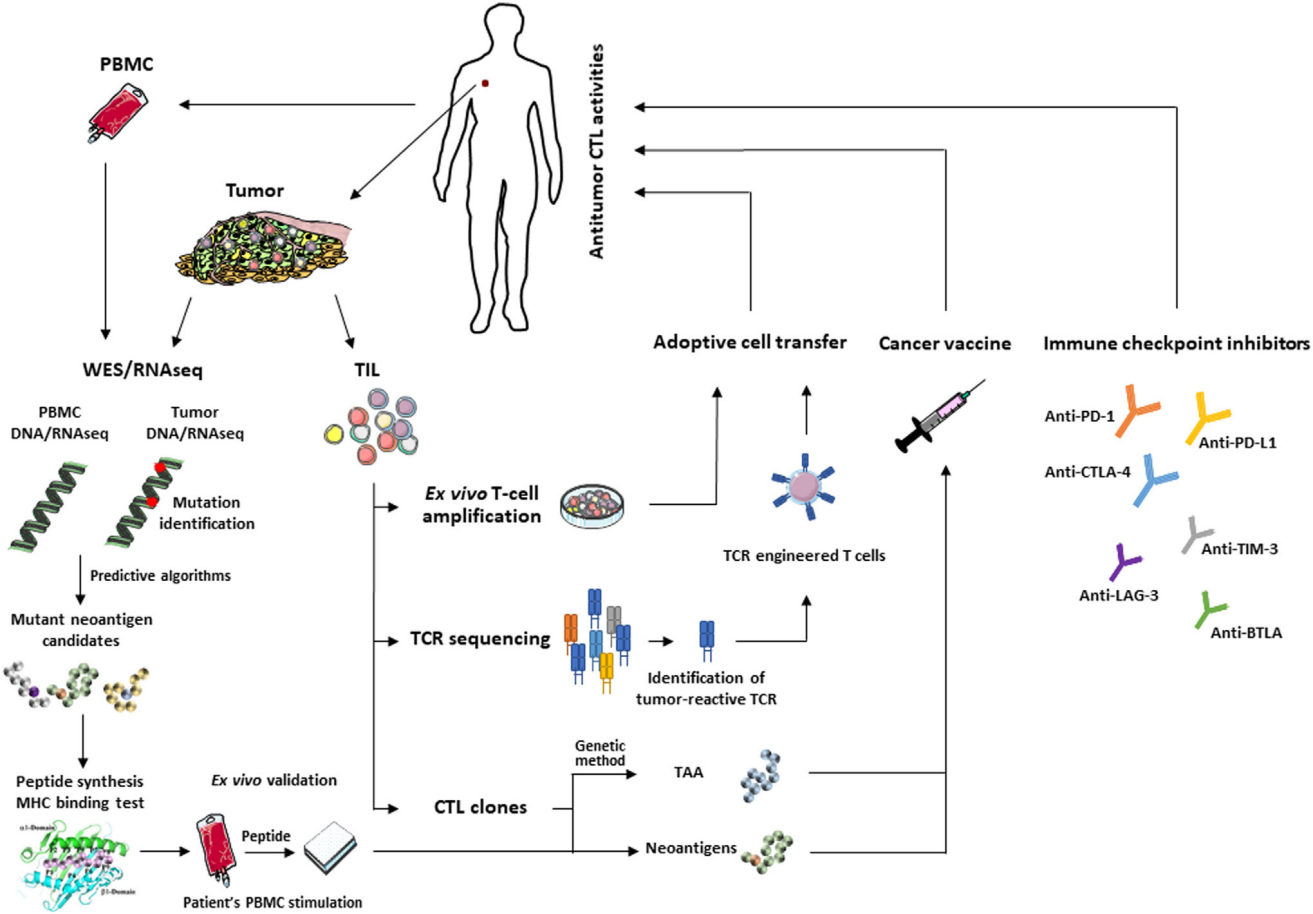
Main approaches for T-cell-based cancer immunotherapy: identification of immunogenic tumor antigens using WES/RNAseq and predictive programs (left) or CTL and genetic approach for the development of effective therapeutic cancer vaccines. Adoptive transfer of selected tumor-infiltrating lymphocytes (TIL) or autologous T cells engineered to express tumor-reactive T-cell receptor (TCR) (middle). These approaches can be combined with immune checkpoint inhibitors (right) to reverse T-cell exhaustion and optimize antitumor T-cell response.

The current challenge in developing more efficient second-generation cancer vaccines is based on mutant epitopes that derive from tumor neoantigens (192, 193). Non-mutant tumor neoepitopes that emerge on the target cell surface upon alteration of TAP expression, such as the self-epitope derived from the human ppCT preprohormone (94, 95), are interesting targets for peptide-based vaccination against immune-escaped tumors expressing low levels of pMHC-I complexes (194, 195). Recent technological advances in identifying mutation-derived tumor antigens have enabled development of patient-specific therapeutic vaccines, including peptides, proteins, DC, tumor cells, and viral vectors, that target individual cancer mutations (196). Over the past few years, examples of TSA-based personalized cancer immunotherapies have begun to emerge. For example, a durable clinical response to cancer vaccines with autologous melanoma-pulsed DC was obtained and correlated with the presence of effector memory T cells responding to mutant antigens (54). Moreover, DC-based vaccination directed at melanoma-neoepitope candidates resulted in an increase in clonal diversity of antitumor T-cell immunity and promoted a diverse neoantigen-specific TCR repertoire (55). Immunogenic personal neoantigen vaccines, based either on RNA or synthesized long peptides, have recently been developed for patients with melanoma. In this regard, personalized RNA-based mutanome vaccines, alone or in combination with anti-PD-1, induced effective T-cell responses against multiple vaccine neoepitopes and resulted in sustained progression-free survival (84). In another clinical trial, long peptide cancer vaccines that target predicted personal tumor neoantigens, administered alone or in combination with anti-PD-1, resulted in clinical benefits and induced polyfunctional CD4^+^ and CD8^+^ T cells, with expansion of the repertoire of neoantigen-specific T cells (132). Thus, a combination of neoepitope-based vaccines and ICI is promising for overcoming the anergic state of vaccine-induced T cells. These strategies open up new avenues for further development of personalized active immunotherapy, either alone or in combination with other therapies, for patients with different types of cancer (Figure 1). Personalized cancer immunotherapies offer promise of low toxicity and high specificity, and the opportunity to treat human malignancies resistant to current therapies.

## Concluding Remarks

The success of cancer immunotherapy relies on the induction of immune effector mechanisms associated with generation of high-avidity tumor-specific CTL. To further improve their antitumor effectiveness, and for more robust long-term disease control, a deeper understanding of host-tumor interactions and tumor immune escape strategies is required. Overcoming immune tolerance/suppression pathways within the tumor microenvironment, which may hinder the potency of immunotherapeutic approaches, is a major challenge in the field of tumor immunology and immunotherapy. In this context, optimizing the therapeutic potential of the immune system relies on a combination of different approaches, mainly cancer vaccines with ICI and/or ACT, which synergistically enhance antitumor T-cell responses. Selection of the right adjuvant or neoadjuvant, such as TLR agonists, is necessary to improve the immunogenicity of peptide-based vaccines, by targeting antigens to competent APC (and, in particular, DC, capable of cross-presentation and delivering of stimuli to activate both specific CD4^+^ and CD8^+^ T cells). Moreover, alternative routes of peptide administration for improved target delivery would help to induce strong long-lasting antitumor T-cell responses and thus improve clinical outcome. Therapeutic cancer vaccines combining both TAP-dependent and TAP-independent epitopes might also boost tumor-specific CD8 T-cell immunity, prevent immune escape mechanisms developed by malignant cells, and thereby potentiate current cancer immunotherapies. Remarkably, targeting of non-self tumor-specific neoantigens, generated by somatic mutations, has gained increasing interest over the past few years. Rising accessibility to NGS technologies, improved *in silico* prediction of truly immunogenic mutant peptides and easy peptide manufacturing are promising approaches to identifying patient-specific neoepitopes and evaluating their potential use in both prognosis and treatment. The utility of highly immunogenic neoantigens for personalizing therapeutic cancer vaccines will open up new perspectives for the refinement of current cancer immunotherapies.

## Author Contributions

FMC, AD, and SC: design and writing. YV: writing.

## Conflict of Interest Statement

The authors declare that the research was conducted in the absence of any commercial or financial relationships that could be construed as a potential conflict of interest.

## Abbreviations

ACT, adoptive cell transfer; CDR, complementarity-determining region; CTL, cytotoxic T lymphocyte; CTLA, cytotoxic T-lymphocyte associated antigen; PD, programmed cell death; DC, dendritic cell; HLA, human leukocyte antigen; ICAM-1, intercellular adhesion molecule 1; ICI, immune checkpoint inhibitors; IFN, interferon; LFA-1, lymphocyte-function-associated antigen-1; mAb, monoclonal antibody, NSCLC, non-small-cell lung carcinoma; MHC-I/β2m, major histocompatibility complex class I/beta-2-microglobulin; TAA, tumor-associated antigen; TCR, T-cell receptor; TIL, tumor-infiltrating lymphocyte; TSA, tumor-specific antigen.
